# Supplementary material for: In vitro self-replication and multicistronic expression of large synthetic genomes
Source: Nat Commun. 2020 Feb 14;11:904. doi: 10.1038/s41467-020-14694-2 (PMC7021806; doi:10.1038/s41467-020-14694-2)
Supplement: Supplementary file 1 — Description of Additional Supplementary Files [file 41467_2020_14694_MOESM1_ESM.docx]

**Title:** Supplementary Data 1

**Description:** MaxQuant output table containing a concise non-redundant list of identified peptide sequences in PURErep expression experiments.

**Title:** Supplementary Data 2

**Description:** MaxQuant output table containing a comprehensive list of the identified proteins in PURErep expression experiments including their heavy isotope labelling statistics.
