# Supplementary material for: In vitro self-replication and multicistronic expression of large synthetic genomes
Source: Nat Commun. 2020 Feb 14;11:904. doi: 10.1038/s41467-020-14694-2 (PMC7021806; doi:10.1038/s41467-020-14694-2)
Supplement: Supplementary file 2 — Supplementary Information [file 41467_2020_14694_MOESM2_ESM.docx]

**Supplementary Information**

**for**

*In vitro* self-replication and multicistronic expression of large synthetic genomes

Libicher et al.

**Supplementary Table 1.** Final concentrations of reagents present in the PURErep 10x energy mix

| compound | value | unit |
| --- | --- | --- |
| 20 natural L-amino acids | 3.6 each | mM |
| Potassium-glutamate | 700 | mM |
| Spermidine | 3.75 | mM |
| Creatine-phosphate potassium salt | 250 | mM |
| E. coli tRNA | 5.18 | g / L |
| HEPES∙KOH pH 8.0 | 1000 | mM |
| Hemi-magnesium glutamate | 79 | mM |
| Dithiothreitol | 60 | mM |

**Supplementary Table 2**. Approximated final protein concentrations in PURErep (based on the protein concentrations of the original PURE system reported by Kuruma and Ueda^1^, which were shown to provide a good estimate for the protein concentrations in the commercial PURExpress system^2^). The table also lists the coding plasmids of each gene used in the TTcDR experiments in this study.

| Enzyme mix (1x) | µg/ml | kDa | nM | encoding plasmid |
| --- | --- | --- | --- | --- |
| EF-Tu | 200 | 43.3 | 4600 | pEFTu |
| EF-Ts | 100 | 31.2 | 3200 | pLD2 |
| IF1 | 20 | 9.1 | 2200 | pLD2 and pEFTu |
| Ala RS | 137.6 | 96.9 | 1400 | pLD2 |
| EF-G | 100 | 78.4 | 1300 | pLD3 |
| MTF | 40 | 35 | 1100 | pLD2 |
| IF3 | 20 | 21.4 | 930 | pLD2 |
| RRF | 20 | 21.5 | 930 | pLD3 |
| PheRS (α+β) | 33 | 37.7 + 87.4 | 260 | pLD2 |
| AsnRS | 44 | 53.4 | 820 | pLD2 |
| IF2 | 80 | 98.2 | 810 | pLD3 |
| IleRS | 79 | 105.1 | 750 | pLD2 |
| RF1 | 20 | 41.4 | 480 | pLD1 |
| RF2 | 20 | 42.1 | 480 | pLD3 |
| GluRS | 25 | 54.7 | 460 | pLD3 |
| RF3 | 20 | 60.3 | 330 | pLD3 |
| ProRS | 20 | 64.5 | 310 | pLD3 |
| AK1 | 6 | 22.8 | 260 | pAK1 |
| AspRS | 16 | 66.8 | 240 | pLD3 |
| LysRS | 13 | 58.4 | 220 | pLD1 |
| T7RNAP | 20 | 99.8 | 200 | pT7pol |
| GlyRS (α+β) | 19 | 34.8 + 77.6 | 170 | pLD3 |
| ThrRS | 12.6 | 74.8 | 170 | pLD1 |
| CK | 8 | 47.8 | 170 | pCKM |
| NDK | 2.2 | 16.4 | 130 | pNDK |
| GlnRS | 7.6 | 64.3 | 120 | pLD1 |
| PPI | 2 | 20.2 | 100 | not used in this study |
| LeuRS | 8 | 98.1 | 80 | pLD1 |
| SerRS | 3.8 | 49.2 | 80 | pLD1 |
| ArgRS | 4 | 65.6 | 60 | pLD1 |
| TrpRS | 2.2 | 38.3 | 60 | pLD1 |
| MetRS | 4.2 | 77.1 | 50 | pLD1 |
| CysRS | 2.4 | 53 | 45 | pLD1 |
| HisRS | 1.6 | 47.9 | 30 | pLD1 |
| TyrRS | 1.2 | 48.8 | 25 | pLD1 |
| ValRS | 1.6 | 109 | 15 | pLD1 |

**Abbreviations**: RS – tRNA synthetase, EF – elongation factor, IF – initiation factor, RF – release factor, MTF – Methionyl-tRNA-formyltransferase, RRF – ribosome recycling factor, AK1 – Adenylate kinase, T7RNAP – T7 RNA-polymerase, CK – Creatine kinase, NDK – Nucleoside-diphosphate-kinase, PPI – Peptidoprolyl-isomerase

**Supplementary Table 3**. Primers used in this study.

| Number | Name | Sequence (5’ - 3’) | Use |
| --- | --- | --- | --- |
| 79 | pREP-qPCR_fw | AGGGTATGGGCGTATGGTTATATG | qPCR |
| 80 | pREP-qPCR_rv | TGTCCCATGCGAGATATGATCG | qPCR |
| 85 | rRNA_fw | GGGCACTCGAAGATACGG | *rrnB* cloning + qPCR |
| 86 | rRNA_rv | CTCGAGCGTTAACTCGAGGC | *rrnB* cloning |
| 134 | pLD1-qPCR_fw | GCATGAACGATTACCTGCCTG | qPCR |
| 135 | pLD1-qPCR_rv | GTAACCGTAGCTGCCGAGC | qPCR |
| 136 | pLD2-qPCR_fw | GGCCGTGTAGCCGTTGAC | qPCR |
| 137 | pLD2-qPCR_rv | CGAGGAAGGAGATGCCAGC | qPCR |
| 138 | pLD3-qPCR_fw | CGCGATATGGCGACCGG | qPCR |
| 139 | pLD3-qPCR_rv | GTTAGAGTCAAGCGGCAGAAC | qPCR |
| 155 | EF-Tu-qPCR_fw | GCAGAACCACGAACGATCG | qPCR |
| 156 | EF-Tu-qPCR_rv | GCGCGATCCTGGTAGTTG | qPCR |
| 91 | rrnB_qPCR_fw | TGCCTGGCGGCCTTAG | qPCR |
| 151 | IF-1_fw | ATGCACCACCACCACCACCACGCGAAAGAAGATAATATTG | cloning of pEFTu |
| 152 | iF-1_rv | TTAGCGCGAGCGGAAGACGATGCG | cloning of pEFTu |
| 153 | pIVEX_His-Tag_rv | GTGGTGGTGGTGGTGGTGCATATGTGCCATGGTATATCTCC | cloning of pEFTu |
| 154 | pIVEX-IF-1_fw | CGCATCGTCTTCCGCTCGCGCTAAAAGGGCGAATTCCAGC | cloning of pEFTu |
| 157 | T7P-EF-Tu_fw | CGATCTTCCCCATCGGCGCCGGTGATGCC | cloning of pEFTu |
| 158 | pET_upstream_fw | TGATGTCGGCGATATAGG | cloning of pEFTu |
| 159 | T7P-EF-Tu_rv | TACGTTCAAACTTTTCTTTAGACATATGTGCCATGGTATATCTCC | cloning of pEFTu |
| 160 | EF-Tu_fw | GGAGATATACCATGGCACATATGTCTAAAGAAAAGTTTGAACGTAC | cloning of pEFTu |
| 161 | EF-Tu_rv | GGCAGCAGCCAACTCTTACCCCAGAACTTTTGCTACAACGCC | cloning of pEFTu |
| 162 | EF-Tu-T7T_fw | GTAGCAAAAGTTCTGGGGTAAGAGTTGGCTGCTGCCA | cloning of pEFTu |
| 163 | T7T-upstr-IF1_rv | CCTATATCGCCGACATCAGGAGCCACTATCGACTACGCG | cloning of pEFTu |
| 200 | Amp_fw | GTCTCATGAGCGGATAC | deletion of AmpR |
| 201 | Amp_rv | AGATCGCTGAGATAGGTG | deletion of AmpR |

| plasmid | gene cargo | size (kb) | reference |
| --- | --- | --- | --- |
| pLD1 | *trpS, lysS, cysS, valS, argS, tyrS, glnS, hisS, leuS, metG, serS, prfA, thrS (E. coli)* | 30.1 | [5] |
| pLD2 | *infA, fmt, infC, pheT, tsf, alaS, pheS, ileS, asnS (E. coli)* | 20.0 | [5] |
| pLD3 | *glyQ, aspS, prfB, glyS, gltX, infB, frr, fusA, proS, prfC (E. coli)* | 23.4 | [5] |
| prRNA | *rrnB (E. coli)* | 8.9 | this work |
| pEFTu | *tufA, infA* (E. coli)* | 5.2 | this work |
| pREP | *gp2 (bacteriophage φ29)* | 4.5 | this work |
| pNDK | *ndk (E. coli)* | 3.0 | this work, derived from Addgene plasmid #124136 |
| pCMK | *CKM (Gallus gallus)* | 3.8 | this work, derived from Addgene plasmid #124134 |
| pAK1 | *AK1 (G. gallus)* | 5.95 | Addgene plasmid #124134 |
| pIPP | *IPP1 (Saccharomyces cerevisiae)* | 6.2 | Addgene plasmid #118978 |
| pT7pol | *p07 (bacteriophage T7)* | 5.3 | this work, derived from Addgene plasmid #124138 |

**Supplementary Table 4**. Plasmids used in this study.

**
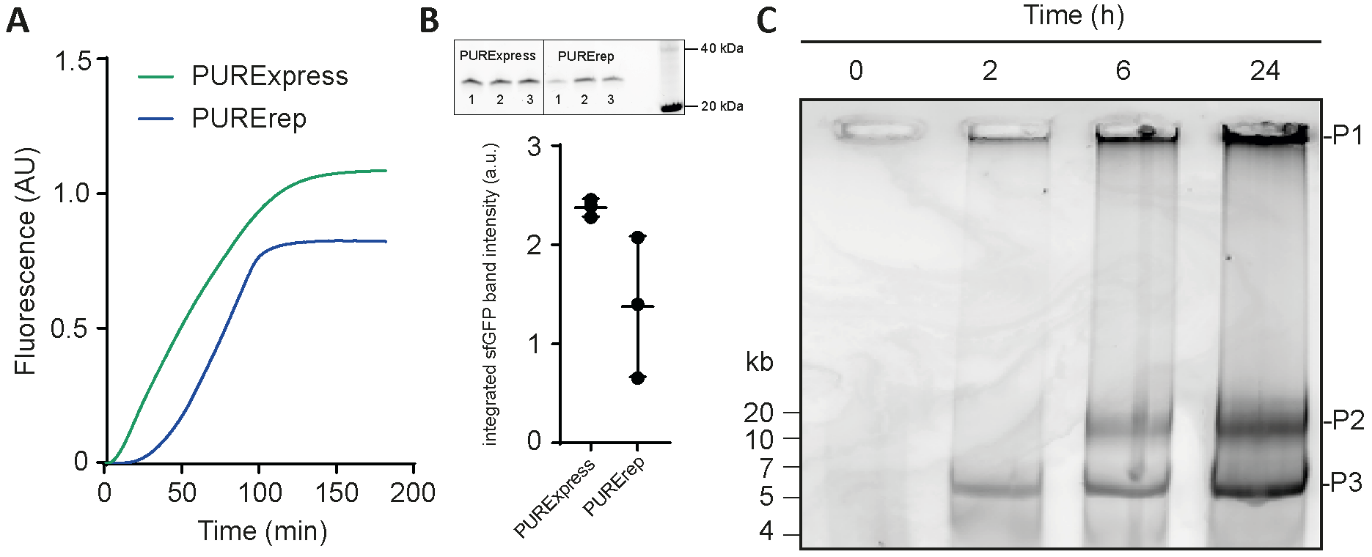
**

**Supplementary Figure 1. Protein expression and DNA replication in the new PURErep system.**

(A) Example comparison of GFP expression in PURExpress (green) and PURErep (blue). Both IVT systems were programmed with the same amount of pIVEX_sfGFP plasmid (4 nM). PURExpress exhibits a higher protein yield as well as a faster onset of protein production. sfGFP expression in PURErep follows a sigmoidal trend with a pronounced lag phase. The different curve shapes might reflect the lower initial rNTP concentrations in PURErep, which result probably result in delayed transcription in PURErep relative to PURExpress. (B) Independent biological triplicates of sfGFP expression in PURExpress and PURErep analysed by PAGE. PURErep typically reaches ~60-80% of PURExpress GFP expression even though sometimes poor batches can have decreased activities of only about 30% of a typical PURExpress batch (exemplified in PURErep, sample 1). Data are presented as mean values +/- stdev (C) Agarose gel electrophoresis of untreated pREP TTcDR samples at different time points of the TTcDR reaction in PURErep (8 nM pREP input concentration). Note that the pREP version used in this experiment encoded a resistance gene for kanamycin zeocin (total plasmid size: 5.4 kb). A considerable amount of stained material was retained in the gel pockets and either represents large-molecular weight rolling-circle concatemer products or DNA adsorbed to MgPP_i_ precipitates^3^ (product 1 – P1). A poorly defined band at ~20 kb (P2) likely reflects lower molecular weight concatemer products of pREP. The time-dependent increase of a DNA species with an apparent size of ~5 kb (P3) suggests that the TTcDR reaction produces significant amounts of unit length plasmid-like products.


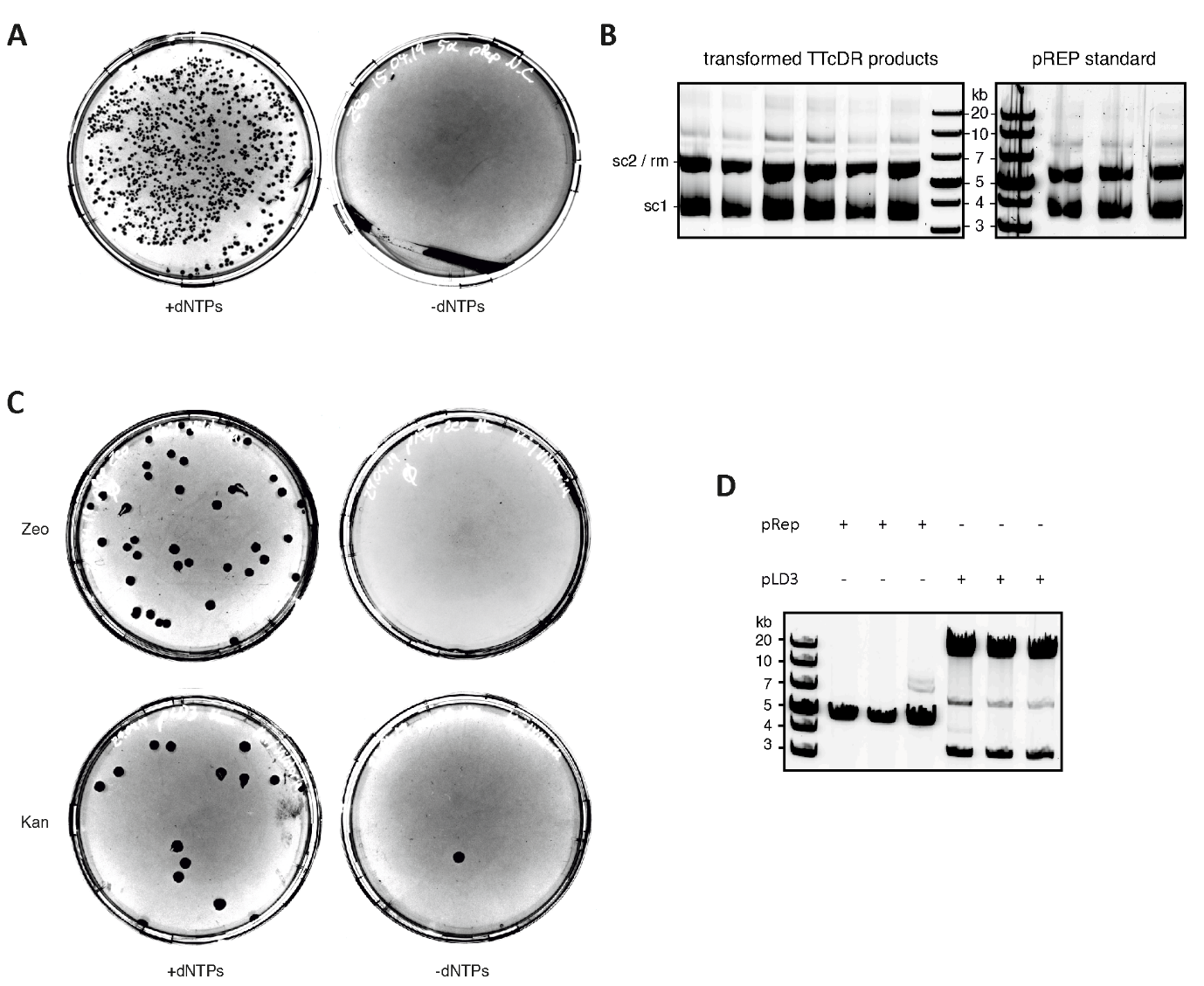


**Supplementary Figure 2. TTcDR reaction products can be transformed into *E. coli* cells where they propagate as circular plasmids.**

**(A)** LB/zeocin plates after transformation of Dpn1-digested pREP TTcDR product into electro-competent *E. coli* 10-beta cells (left plate: +dNTPs, right plate: -dNTPs). **(B)** Representative agarose gel of untreated plasmids purified from randomly picked colonies (left gel). The two major bands are likely to reflect supercoiled monomers (sc_1_) and supercoiled dimers (sc2) or relaxed monomers (rm)^4^. The gel to the right shows equivalent samples from preparations of pREP plasmids, which were exclusively propagated *in vivo*. Samples were tested in biological replicates (n = 5). **(C)** Transformation of pREP / pLD3 co-replication reactions after Dpn1-digest. LB-plates were either selective for pREP (Zeo - zeocin, top plates) or pLD3 (Kan - kanamycin, lower plates). TTcDR reactions were either carried out in presence of NTP (+dNTP) or, as negative controls, in absence of dNTPs (-dNTP). **(D)** Representative Mlu1-digest of plasmids purified from randomly picked colonies from either LB/zeocin or LB/kanamycin plates from +dNTP reactions. The restriction fingerprints correspond to the expected restriction patterns of pREP and pLD3 respectively. Samples were tested in biological triplicates.


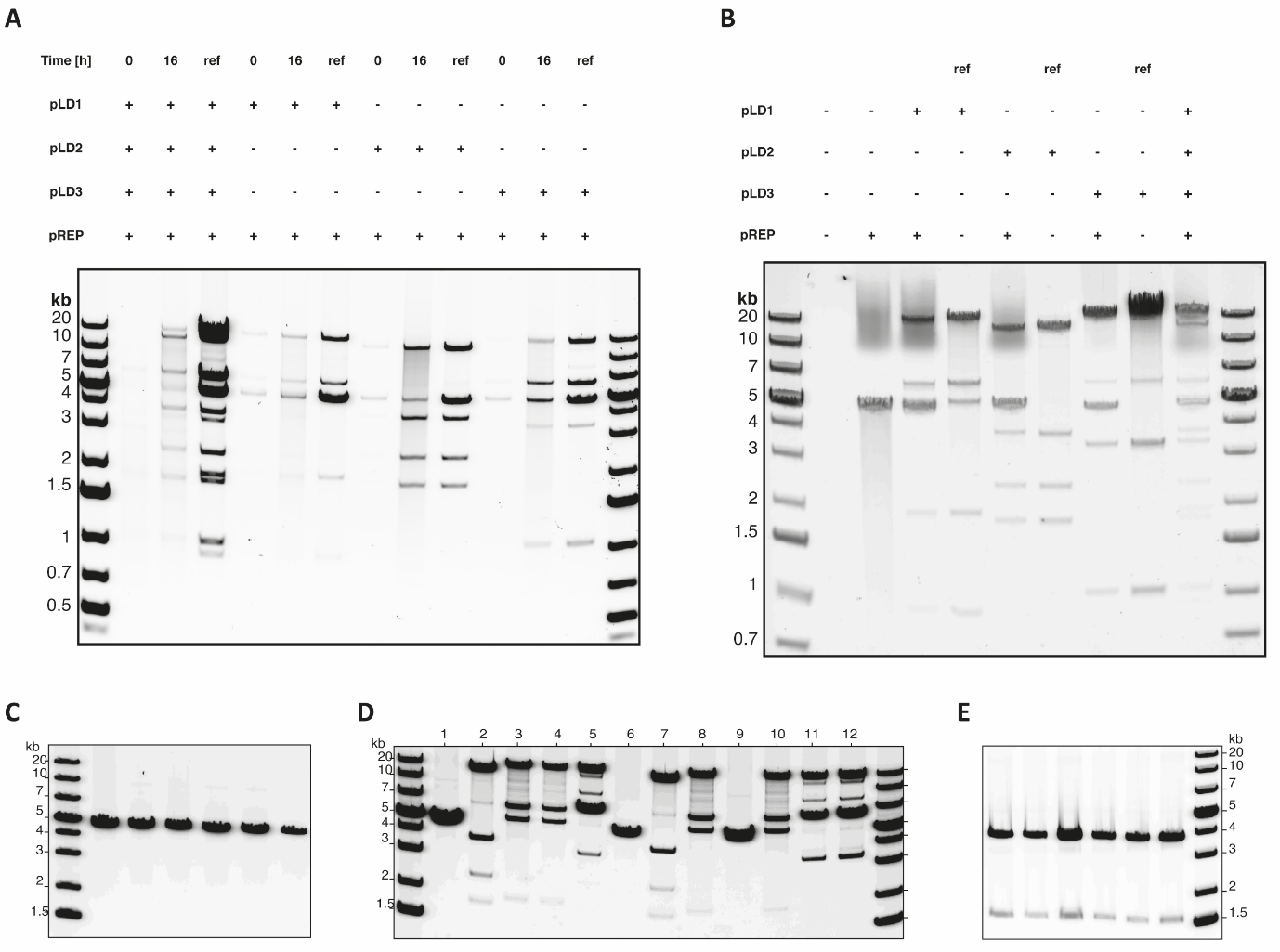


**Supplementary Figure 3. Co-replication of two, four and six plasmids can be confirmed by restriction pattern analysis.**

**(A)** Representative uncropped gel images of the MluI restriction patterns of gel-purified TTcDR products from individual pLD/pREP co-replication experiments at t = 0 h and t = 16 h shown in Figure 2A. Input plasmid concentrations were 6 nM pREP and 0.7 nM pLD1, pLD2 or pLD3. Dpn1 was used to eliminate parental plasmid background. Authentic reference standards for clonal pREP / pLD mixtures are shown for each TTcDR reaction (ref). **(B)** Representative uncropped gel image to the pLD MluI fingerprints shown in Figure 2B. n: negative control / no dNTP, p: pREP only, ref: purified plasmid reference. Input concentrations of plasmids were 2 nM of each pLD plasmid (lane 3, 5, 7) and 4 nM pREP. Input concentrations for the co-replication of all three pLD plasmids (lane 8) were 4 nM pREP and 2 nM pLD1-3. **(C, D, E)** Representative MluI restriction digests of plasmid samples purified from randomly picked colonies from the plates shown in Figure 2D. Experiments were repeated in independent experiments to ensure reproducibility (n = 2 to 5). (C) LB/zeocin plate: all restriction patterns correspond to pREP (4.6 kb). (D) MluI digested plasmids isolated from colonies grown on the LB/kanamycin plate. Lanes 1, 6 and 9: prRNA; lanes 2 and 7: pLD2; lanes 5, 11 and 12: pLD1; lanes 3, 4, 8 and 10: pLD3. (E) LB/Cb plate, XbaI-digested plasmids. All restriction patterns correspond to pEFTu.

**
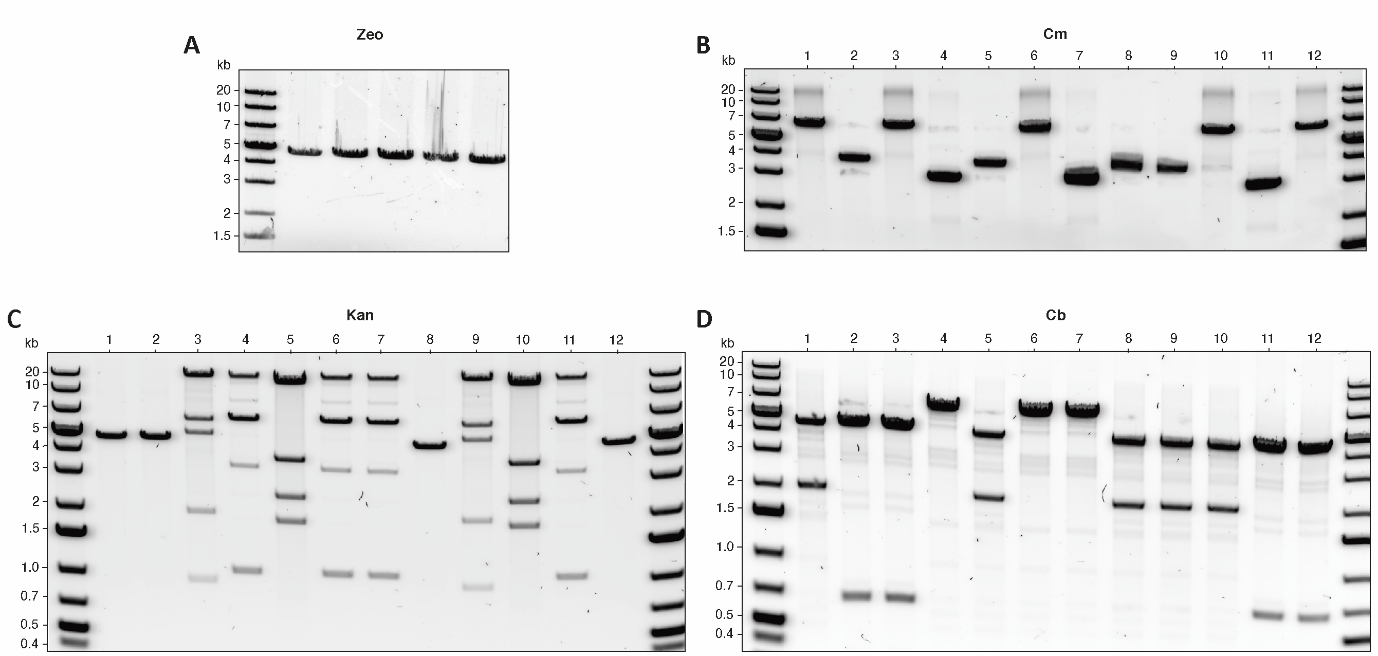
**

**Supplementary Figure 4. Co-replication of 11 plasmids confirmed by restriction pattern analysis.**

Representative restriction analyses of plasmids samples purified from randomly picked colonies from the plates shown in Figure 3B. Samples were tested in biological replicates to ensure reproducibility (n = 2 - 3). **(A)** LB/zeocin plate: all restriction patterns correspond to pREP (4.6 kb) digested by MluI **(B)** XbaI restriction patterns for plasmids isolated from colonies grown on LB/chloramphenicol. Restriction patterns match pT7pol (lanes 1, 3, 6, 10, 12), pCMK (lanes 2, 5, 8, 9) and pNDK (lanes 4, 7, 11). **(C)** MluI restriction patterns for plasmids isolated from colonies grown on LB/kanamycin plates. Restriction patterns match prRNA (lanes 1, 2, 8, 12), pLD1 (lanes 3 and 9) and pAK1 (lanes 5 and 10), pLD3 (lanes 4, 6, 7, 11). **(D)** EcoRV restriction patterns for plasmids isolated from colonies grown on LB/ carbenicillin plates. Restriction patterns match pIPP (lanes 1, 5, 8, 9, 10), pEFTu (lanes 2, 3, 11, 12) and pAK1 (lanes 4, 6, 7).


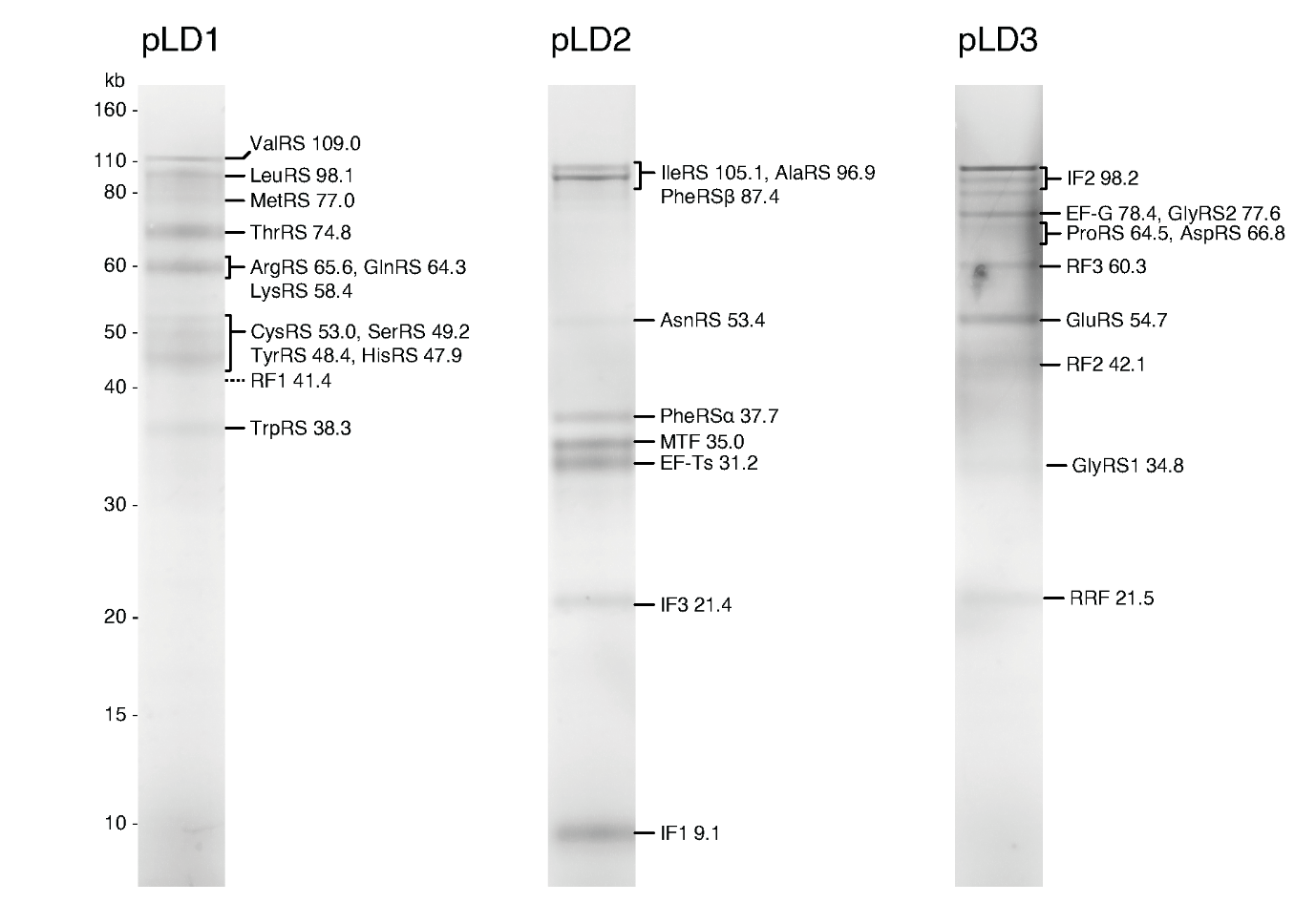


**Supplementary Figure 5. *De novo* synthesis of pLD-encoded translation factors in PURErep.**

Representative GreenLys-based detection of the 30 pLD-encoded *E. coli* translation factors (32 protein subunits) after translation in PURErep. Experiment was repeated twice with similar results. Proteins were annotated according to their expected size (Table S2) and the band patterns shown in Sheperd *et al*.^5^ Please note that band intensities does not necessarily correlate with expression levels since fluorescence levels are dependent on the number of lysine residues present in the individual protein sequences.

**Supplementary References**

1. Kuruma, Y. & Ueda, T. The PURE system for the cell-free synthesis of membrane proteins. *Nat. Protoc.* **10**, 1328–1344 (2015).

2. Lavickova, B. & Maerkl, S. J. A Simple, Robust, and Low-Cost Method To Produce the PURE Cell-Free System. *ACS Synth. Biol.* **8**, 455–462 (2019).

3. Galinis, R. *et al.* DNA Nanoparticles for Improved Protein Synthesis In Vitro. *Angew. Chemie* **128**, 3172–3175 (2016).

4. Munson, B. R., Hucul, J. A., Maier, P. G., Krajewski, C. A. & Helmstetter, C. E. E. coli minichromosome replication in vitro and in vivo: comparative analyses of replication intermediates. *Biochim. Biophys. Acta - Gene Struct. Expr.* **910**, 11–20 (1987).

5. Shepherd, T. R. *et al.* De novodesign and synthesis of a 30-cistron translation-factor module. *Nucleic Acids Res.* **45**, 10895–10905 (2017).
