# Supplementary material for: In vitro self-replication and multicistronic expression of large synthetic genomes
Source: Nat Commun. 2020 Feb 14;11:904. doi: 10.1038/s41467-020-14694-2 (PMC7021806; doi:10.1038/s41467-020-14694-2)
Supplement: Supplementary file 6 — Reporting Summary [file 41467_2020_14694_MOESM6_ESM.pdf]

nature research

Corresponding author(s): Hannes Mutschler

Last updated by author(s): Jan 23, 2020

Reporting Summary

Nature Research wishes to improve the reproducibility of the work that we publish. This form provides structure for consistency and transparency in reporting. For further information on Nature Research policies, see Authors & Referees and the Editorial Policy Checklist .

Statistics

For all statistical analyses, confirm that the following items are present in the figure legend, table legend, main text, or Methods section.

n/a	Confirmed	
☐	☒	The exact sample size (*n*) for each experimental group/condition, given as a discrete number and unit of measurement
☐	☒	A statement on whether measurements were taken from distinct samples or whether the same sample was measured repeatedly
☒	☐	The statistical test(s) used AND whether they are one- or two-sided<br>*Only common tests should be described solely by name; describe more complex techniques in the Methods section.*
☒	☐	A description of all covariates tested
☒	☐	A description of any assumptions or corrections, such as tests of normality and adjustment for multiple comparisons
☐	☒	A full description of the statistical parameters including central tendency (e.g. means) or other basic estimates (e.g. regression coefficient) AND variation (e.g. standard deviation) or associated estimates of uncertainty (e.g. confidence intervals)
☒	☐	For null hypothesis testing, the test statistic (e.g. *F*, *t*, *r*) with confidence intervals, effect sizes, degrees of freedom and *P* value noted<br>*Give P values as exact values whenever suitable.*
☒	☐	For Bayesian analysis, information on the choice of priors and Markov chain Monte Carlo settings
☒	☐	For hierarchical and complex designs, identification of the appropriate level for tests and full reporting of outcomes
☒	☐	Estimates of effect sizes (e.g. Cohen's *d*, Pearson's *r*), indicating how they were calculated

Our web collection on statistics for biologists contains articles on many of the points above.

Software and code

Policy information about availability of computer code

Data collection	StepOne™ and StepOnePlus™ Software v2.3, Typhoon Scanner Control 5.0, ImageJ 1.51i
Data analysis	LinRegPCR 2018.0, MaxQuant version 1.6.6.0, ImageQuant TL 8.1, Graphpad Prism 7.05

For manuscripts utilizing custom algorithms or software that are central to the research but not yet described in published literature, software must be made available to editors/reviewers. We strongly encourage code deposition in a community repository (e.g. GitHub). See the Nature Research guidelines for submitting code & software for further information.

Data

Policy information about availability of data

All manuscripts must include a data availability statement. This statement should provide the following information, where applicable:

- Accession codes, unique identifiers, or web links for publicly available datasets
- A list of figures that have associated raw data
- A description of any restrictions on data availability

The source data underlying Figs. 1B, 1C, 1E, 1F, 2A, 2B, 2C, 2E, 3B, 4A, 4B, Supplementary Figs. S1B, S1C and 5 are provided in a Source Data file. MaxQuant outputs are provided in two separate source files. Further data supporting the findings of this paper are available from the corresponding author upon reasonable request.

Field-specific reporting

Please select the one below that is the best fit for your research. If you are not sure, read the appropriate sections before making your selection.

☒ Life sciences ☐ Behavioural & social sciences ☐ Ecological, evolutionary & environmental sciences

Life sciences study design

All studies must disclose on these points even when the disclosure is negative.

Sample size	Typical sample sizes were n=3 to 5 biological replicates. In qPCR experiments each biological replicate was determined from the mean of a technical triplicate. Sample sizes were determined based on previous experience and published data to evaluate the reproducibility. No statistical methods were used to determine the sample size.
Data exclusions	A small subfraction of Cq-values resulting from qPCR traces did not pass the quality filters of the LinReg software package and were excluded from further analysis. Exclusion criteria reported by the software were either the absence of a defined plateau or PCR efficiency outside 10%. The excluded Cq values are shown in the source data file (red cells). H/L ratios from 2 out of 16 isotope labeling experiments were from compromised PURErep batches and showed H/L counts of less than 15%-20% compared to other independent replicates from fully active PURErep batches. The excluded datasets are included in the MaxQuant output spreadsheets Orbi3366peptides.xlsx and Orbi3366proteinGroups.xlsx.
Replication	Experimental findings were reliably reproduced using different PURE batches, plasmid preparations and chemical stock solutions.
Randomization	Not applicable to this study because group allocation was not carried out.
Blinding	Not applicable to this study because group allocation was not carried out.

Reporting for specific materials, systems and methods

We require information from authors about some types of materials, experimental systems and methods used in many studies. Here, indicate whether each material, system or method listed is relevant to your study. If you are not sure if a list item applies to your research, read the appropriate section before selecting a response.

Materials & experimental systems

n/a	Involved in the study	
☒	☐	Antibodies
☒	☐	Eukaryotic cell lines
☒	☐	Palaeontology
☒	☐	Animals and other organisms
☒	☐	Human research participants
☒	☐	Clinical data

Methods

n/a	Involved in the study	
☒	☐	ChIP-seq
☒	☐	Flow cytometry
☒	☐	MRI-based neuroimaging
